# Candies

**Published:** 1871-06

**Authors:** 


### CANDIES.

It is impossible for pure candies to injure the teeth by anything they leave on or about them, because they are perfectly dissolved before swallowing, and have no sediment or residue. Candies, like cheese and nuts, taken as the sole dessert, after a meal, promote digestion; they are only injurious by being taken largely between meals; but they are more appropriate in cold weather. The best desserts for summer, are the fruits in their season, in their natural, fresh, perfect state, because candies are warming, and fruits are cooling; their acid seeming to have the effect of separating the bile from the blood; and fevers are caused by there being too much bile in the blood; therefore "sours" are for the summer, and "sweets" for the winter time; meats are heating, vegetables are cooling; those who are wise, will regulate themselves accordingly.

